# Controlled Arrangement of Gold Nanoparticles on Planar Surfaces via Constrained Dewetting of Surface-Grafted RAFT Polymer

**DOI:** 10.3390/polym12061214

**Published:** 2020-05-26

**Authors:** Katharina Hendrich, Wentao Peng, Philipp Vana

**Affiliations:** Georg-August-University Göttingen, Institute of Physical Chemistry, Tammannstr. 6, D-37077 Göttingen, Germany; katharina.dabow@stud.uni-goettingen.de (K.H.); wentao.peng@stud.uni-goettingen.de (W.P.)

**Keywords:** grafting-to, RAFT polymerization, star polymer, gold nanoparticles, constrained dewetting, nanostructured surfaces

## Abstract

Linear and four-arm star polystyrene samples prepared by RAFT polymerization were grafted to gold surfaces directly via their thiocarbonylthio-end groups. Nanoscale polymer patterns were subsequently formed via constrained dewetting. The patterned polymer films then served as a template for the precise arrangement of gold nanoparticles in a monolayer with a well-defined and regular structure. Using star polymers as a linker between the planar gold surface and the particles, the structural stability of the arranged particles can be further enhanced. The surface-bound nanocomposite films made of polymer and nanoparticles can also reversibly switch their nanostructures by simple wetting or dewetting treatment.

## 1. Introduction

Creating structured surfaces is one of the major challenges in micro- and nanotechnology [1]. In this context, polymeric systems can serve as a smart tool to tune surface properties, for instance, permeability, roughness, wettability or biocompatibility [2]. Numerous approaches to tailor nanostructured polymer surfaces were recently established [3]. Using electron-beam lithography, feature sizes down to 7 nm can be generated [4]. Dip-pen nanolithography, nanoshaving and scanning electrochemical microscope etching are further examples for the fabrication of polymeric nanopatterned surfaces [5,6,7]. One principle feature of polymer layers is their possible response to external stimuli such as temperature, solvent properties or light [8,9]. The response to external stimuli often leads to a conformational change within the polymeric layer which can be exploited for surface-patterning design. For example, block-copolymer brushes can undergo the well-known microphase separation, leading to nanostructured surfaces of high regularity under certain solvent and temperature conditions [10,11]. It is, however, less known, that also surface-bound homopolymers can form very regular surface features when exposed to certain solvent conditions [12,13]. It is tempting to combine this straight-forward approach with nanoparticles in order to further enhance the complexity of such nanostructured surfaces and to expand their area of application.

Hence, nanostructured homopolymer layers with features of several nanometers were targeted in the present work by using polystyrene from reversible addition–fragmentation chain transfer (RAFT) polymerization [14]. RAFT polystyrene, with its aurophilic end-groups, was anchored onto the gold surface [15,16,17] with a low grafting density and allowed to form nanosized patterns under poor solvent conditions, according to the so-called constrained dewetting process. There, polymer chains collapse at the substrate-surface to minimize polymer–solvent interaction [12,13]. The formed polymer aggregates constitute a polymeric nanostructure with different types of nanopatterns, ranging from spherical micelles over wormlike micelles to a network of micelles [13]. The morphology of the nanostructure mainly depends on the grafting density, as nicely demonstrated by Kumacheva and coworkers [18]. 

In the present work, we apply the concept of topology design to the polymer brush to extend the capability of the nanopatterned polymer layer from dewetting. Polystyrene was synthesized via RAFT polymerization, which makes it possible to control both the end-group functionality and the polymer topology, e.g., forming star-shaped polymers [19]. Employing the so-called R-group approach, each arm of the star polymer contains a RAFT functionality at the terminal end that can function as an anchoring site. Our previous work demonstrated the ability of star RAFT polymer to act as a linker between two types of gold nanoparticles (AuNPs) for fabricating planet–satellite nanostructures [20]. In the present work, a secondary layer of AuNPs can be self-assembled at the planar polymer surface by using RAFT-star-polymer. Kumacheva et al. [18] demonstrated that nanoparticles may attach to surfaces with dewetted polymer films, but found that the NPs are densely packed and clustered in the areas where nearly no polymer is present, and that no particles are present in the polymer-rich areas. The aim of the present work was to adjust the strategy of constrained dewetting with subsequent nanoparticle attachment by using RAFT polymerization and specific polymer brush topologies in order to arrive at a surface-bound nanoparticle pattern of much higher regularity, higher stability, and with individual and well-separated nanoparticles being present on the planar surface.

## 2. Materials and Methods

### 2.1. Atomic Force Microscopy and s-SNOM

Atomic force microscopy (AFM) characterization was performed in the dry state using a Multimode 8 AFM (Bruker, Karlsruhe, Germany) with a NanoScope V controller in an ambient environment. Soft probes (ScanAsyst-Air-HR, Bruker) with a nominal spring constant of 0.4 N·m^−1^, a nominal resonance frequency of 70 kHz and a nominal tip radius of 2 nm were used. The ScanAsyst-HR in Air mode was used as the imaging mode, with a scan rate of 0.97 Hz and a resolution of 512 × 512 samples per line. To measure the mechanical properties of the samples, PeakForce-Quantitative Nanomechanical Mapping (PeakForce-QNM) mode was used. 

The depiction of the images was accomplished with the NanoScope Analysis program (Bruker, Karlsruhe, Germany). For the calculation of the Minkowski quantities, the Anaconda software (Python distribution, version 2019.10) was used. For the specification of the formed domains and the classification of the nanostructures, the image processing program ImageJ (version 1.53) was applied.

s-SNOM-measurements were conducted on a neaSNOM (Near-Field Scanning Optical Microscope) by *neaspec*, Munich, Germany, equipped with a near-field imaging system (Optical Detection Modules for Nano-Imaging) including a Near-Field Spectroscopy System (nano-FTIR).

### 2.2. Spectroscopic Ellipsometry

The thicknesses of the thin polymer films were characterized using a spectroscopic ellipsometer Nanofilm EP4 (Accurion, Göttingen, Germany). Measurements were performed using nulling ellipsometry over a wavelength range of 375 to 800 nm with a laser-stabilized xenon arc lamp with grating monochromator at an angle of incidence of light of 50°. The polymer film thickness on glass coverslips with a gold layer was determined at least at three different spots for statistical significance.

### 2.3. Size-Exclusion Chromatography

Size-exclusion chromatography was performed with THF at 35 °C as eluent using an AGILENT 1260 INFINITY system (Santa Clara, CA, USA). The setup comprised an isocratic HPLC-pump and an autosampler, a PSS SDV guard column (8 × 50 mm) and three PSS SDV separation columns (8 × 300 mm, particle size = 5 μm, pore sizes = 10^3^, 10^5^ and 10^6^ Å). As the detection system, an RI detector and an UV detector that was set to a wavelength of 310 nm were used. The flow rate of the mobile phase was 1.0 × 10^−3^ L·min^−1^. Polymer samples were dissolved in THF with toluene as the internal standard and filtered through a syringe filter with a polytetrafluoroethylene (PTFE) membrane with a pore size of 0.45 μm. The concentration of the polymer solutions was 3 g/L. The system was calibrated using PSS polystyrene standards of low dispersity for the measurement of polystyrene samples.

### 2.4. Chemicals

The used solvents, i.e., acetone, dichloromethane, dimethylformamide, ethanol, methanol, tetrahydrofuran, and toluene, were purchased in pro analysi grade. Styrene was freshly purified prior to use by passing through a column of basic aluminum oxide (Brockmann I, 150 mesh, Merck, Darmstadt, Germany). Anhydrous dichlormethane (Merck, Darmstadt, Germany), 3-aminopropyldimethylethoxysilane (ABCR, Karlsruhe, Germany), Atto 655-maleimide (Merck, Darmstadt, Germany), butylamine (Alfa Aesar, Kandel, Germany), 4-cyano-4-[(dodecylsulfanylthiocarbonyl)sulfanyl]pentanoic acid (ABCR, Karlsruhe, Germany), hydrogen tetrachloroaurate trihydrate ABCR), 2-mercapto-2-thiazoline (Merck, Darmstadt, Germany), *N,N*‘-dicyclohexylcarbodiimide (Merck, Darmstadt, Germany), propyldimethoxysilane (ABCR, Karlsruhe, Germany), sodium borohydride (Merck, Darmstadt, Germany), tetra-*N*-octylammonium bromide (ABCR, Karlsruhe, Germany), tris(2-carboxyethyl)phosphine hydrochloride (ABCR, Karlsruhe, Germany), (dimethylamino)-pyridine (MerckDarmstadt, Germany) were purchased in the highest purity available and used as received. Magnesium sulfate (99%, Grüssing, Filsum, Germany) and potassium hydroxide pellets (≥85%, Merck, Darmstadt, Germany) were used as received. Nanopure (type I) water was obtained using a Millipore filtration system equipped with a UV lamp.

### 2.5. Glass Coverslips with Gold Nanolayer

Glass coverslips (Menzel, thickness 150 μm, refractive index 1.52) were placed in a solution of 10 g potassium hydroxide pellets in 50 mL of water and 150 mL ethanol, and then treated with ultrasonication for 10 min. Afterward, the glass coverslips were washed three times with nanopure water and treated with ultrasonication for 5 min. The substrates were dried using argon flow and finally treated with air plasma. The cleaned substrates were used for vapor deposition of 2 nm titanium and 10 nm of gold unless stated otherwise. The evaporation was carried out under high-vacuum conditions (∼10^−6^ mbar) by using an electron beam source (Univex 350, Leybold, Cologne, Germany). Unless stated otherwise, a deposition rate of 1 Å·s^−1^ was used to ensure maximum smoothness on the surface. The thickness of the layers was monitored using an oscillating quartz unit during deposition. The substrates were immediately used or stored. 

### 2.6. Homopolymerization with Styrene

Styrene polymerizations were conducted in bulk and without any initiator via thermal-induced self-initiation. To achieve different molar masses, two polymerization mixtures with the [M]:[RAFT] ratios of 500:1 and 1000:1 were prepared for both linear and star-shaped RAFT agents. All polymerization vials were degassed with argon for at least 5 min and heated to 110 °C for 6 h, 12 h and 24 h, respectively. The reaction was stopped by quenching with oxygen and cooling down with ice water. The free polymer was dissolved in dichloromethane and precipitated in ice-cold methanol to remove any residual monomer. The polymer was separated by centrifugation; two dissolving-centrifugation cycles were performed. The 4-arm star-shaped RAFT agent was synthesized as reported earlier by us in [21]. The molar mass of the obtained polymers is collated in Table 1.

### 2.7. Synthesis of Gold Nanoparticles

Two different kinds of gold nanoparticles (AuNPs) were used in these studies. The citrate-stabilized AuNPs (β(Au) ≈ 0.1 mg·mL^−1^) were obtained as an aqueous solution. The AuNPs from Brust-Schiffrin synthesis were dispersed in toluene. Both AuNPs were synthesized as reported by us in [20]. 

**Figure 1:**
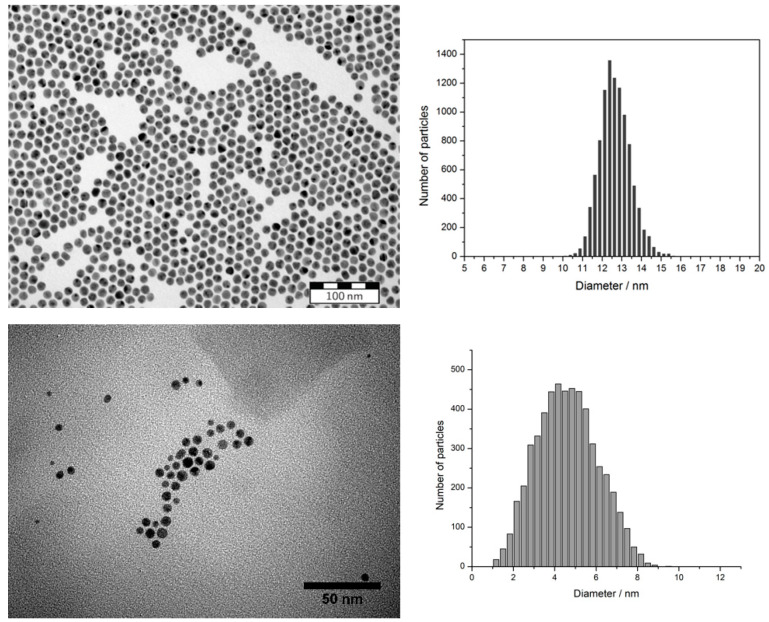
Representative transmission electron micrographs of citrate-stabilized gold nanoparticles (top-left) and AuNPs from Brust-Schiffrin synthesis (bottom-left). The corresponding size distribution of the AuNPs are shown in the figures on the right side.

The average particle size of the respective gold nanoparticles was 5 nm for the tetraoctylammonium bromide-capped NPs from the Brust-Schiffrin synthesis, and 13 nm for the citrate-stabilized NPs. The particle size was determined using transmission electron microscopy (TEM) (see Figure 1).

### 2.8. Immobilization of RAFT-Terminated Polystyrene

Petri dishes were cleaned by sonication with a solution of 10 g potassium hydroxide in 50 mL of water and 150 mL ethanol for 10 min. Afterward, they were washed three times with nanopure water and further sonicated for 5 min. The dishes were dried in vacuo and treated with air plasma. Solutions of RAFT-terminated polystyrene with concentrations ranging from 2 × 10^−3^ mg·mL^−1^ to 2 mg·mL^−1^ in toluene were freshly prepared. The glass coverslips with an evaporated gold layer were then immersed in 2 mL of the polymer solution for various incubation times, ranging from 10 s to 120 s. Afterward, the samples were rinsed with toluene to remove any excess polymer, and dried using argon flow.

### 2.9. Nanostructure Formation

The RAFT polystyrene functionalized substrates were immersed in dimethylformamide in a freshly cleaned petri dish for at least 3 min. The wet substrates were removed and 1 mL nanopure water was cast dropwise onto the surface of the substrates. The gold substrates with nanostructured polystyrene films were gently dried using argon flow. The formed nanostructure was then analyzed via AFM.

### 2.10. Selective Self-Assembly of Gold Nanoparticles

Substrates with tethered polystyrene were treated with the precalculated amount of AuNP colloid (both citrate-capped and Brush-Shiffrin type). For citrate-capped AuNPs, the sol was used as-synthesized (*β*(Au) ≈ 0.1 mg·mL^−1^). The Brust-Schiffrin-type AuNPs were diluted with toluene to the same concentration. After the immersion in the AuNP sol for the predetermined time, i.e., from ten minutes to 24 h, the substrates were removed and rinsed with solvent to remove excess nanoparticles. The nanostructured surfaces were finally dried using argon. 

## 3. Results and Discussion

First, the gold-covered substrates were grafted with RAFT polymer under various conditions. The grafting density of the polymer can be tuned by the concentration of the supernatant solution and by the grafting time. The grafting process was monitored and verified by s-SNOM. The surface-bound polymer was then structured by the constrained dewetting process, which leads to a nanostructured surface. The morphology of the nanostructured surface depends on the grafting density of the tethered polymer, as reported earlier [13,18]. For the calculation of the grafting density, the polymer layer thickness was required, which was determined by nulling ellipsometry.

**Figure 2:**
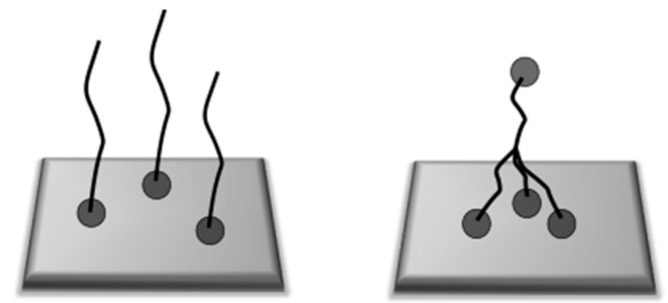
Linear polymer chains with one grafting point (**left**) compared to a four-arm star-shaped polymer with multiple grafting points (**right**).

We further grafted star polymers to gold surfaces using a similar protocol. The star-shaped polymers were synthesized via star RAFT agents using the R-group approach [20]. Through this approach, each arm of the star polymer contains an aurophilic RAFT terminal end-group. It is worth mentioning here that although a star polymer has a smaller radius of gyration compared to the linear polymer with the same molecular weight, the star geometry requires more space on the surface than a linear polymer chain, as illustrated in Figure 2.

### 3.1. Reversibility of the Surface Morphologies

**Figure 3:**
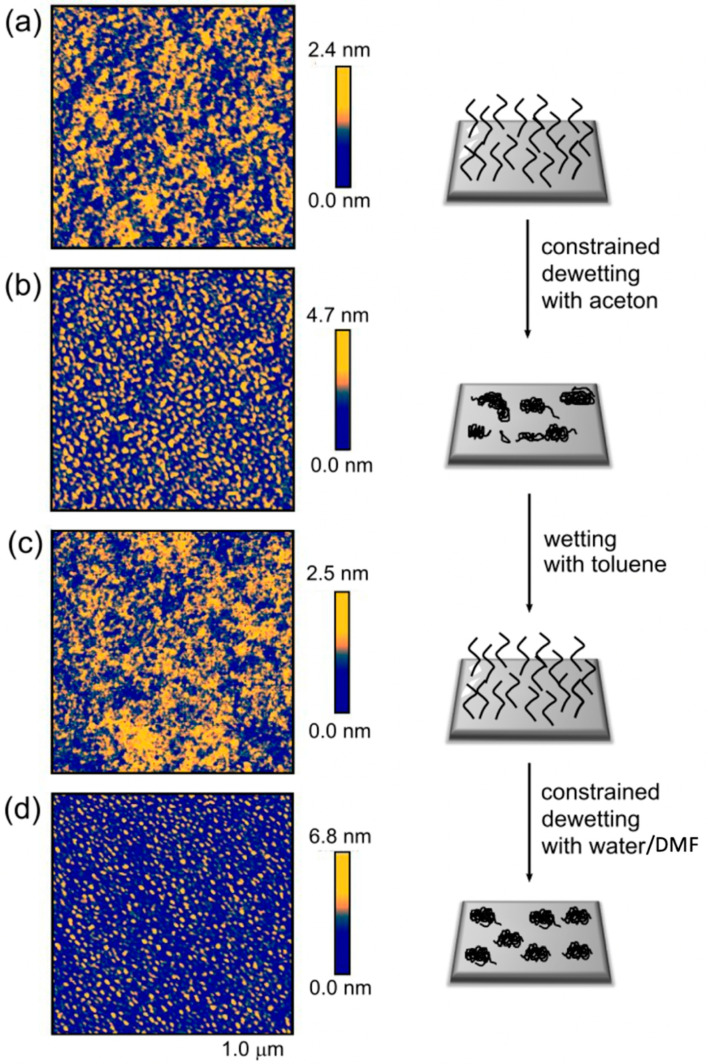
Constrained dewetting of surface-grafted linear polystyrene (*M*_n_ = 64 kg/mol, Σ = 3.6) with two different solvents. (**a**) Homogeneous polymer layer mediated by toluene, (**b**) nanostructure of polystyrene formed by immersion in acetone, (**c**) homogenous polymer layer mediated by toluene, (**d**) nanostructure of polystyrene formed by dewetting with dimethylformamide and water.

In this section, the reversibility and modification of the morphology through different solvent treatments are studied. The obtained nanostructure of polymer on the surface depends on the grafting density and the solvent treatment. Figure 3 shows the nanostructured polymer from a dewetting process of a thin linear polystyrene layer with *M*_n_ = 64 kg/mol and a reduced grafting density of Σ = 3.6 (the reduced grafting density was calculated according to [22] and [8], facilitating comparisons of grafted polymers of different topologies). If the grafted polymer film is immersed in a “good” solvent (e.g., toluene), the polymer chains swell and stretch away from the surface. By drying the substrate, a relatively homogenous polymer film is formed, as indicated by the AFM image shown in Figure 3a. The root mean square roughness, *R*_q_, (root mean square average of the roughness profile ordinates) of these structures was very low, ranging from 0.25 nm to 0.33 nm. The difference in height that is shown in the color bar next to the AFM images varied by around 2.5 ± 0.2 nm, indicating a highly homogeneous morphology.

When the same substrate was immersed again in toluene and the solvent “quality” was subsequently reduced by the addition of acetone, a structured nanopattern of the polymer was formed (see Figure 3b). The AFM image shows an irregular nanostructure consisting of separated micelles and worm-like formations having a domain size of around 20 nm. The roughness *R*_q_ and difference in height increased to 0.66 nm and 4.7 nm, respectively. The nanostructure could be reversibly converted to a homogeneous layer once the substrate was wetted with toluene again (see Figure 3c).

If the substrate was deposited in dimethylformamide and the solvent quality was subsequently reduced by the addition of water, a regular micelle-like nanopattern of the grafted polymer with ca. 15 nm domain size was obtained (see Figure 3d). The constrained dewetting with dimethylformamide and water yielded the highest roughness, i.e., *R*_q_ = 0.94 nm, and a height difference of 6.8 nm, since water is the poorest solvent used here. The nanostructures caused by constrained dewetting of polymer chains tethered to a surface are clearly dependent on the chosen solvent conditions [12,13]. In poor solvents, the polymer chains collapse to avoid polymer-solvent interactions. This is balanced by the tendency of the polymer chains to stretch away from the surface. The poorer the solvent quality, the more prone the polymer chains are to minimizing polymer–solvent interactions, leading to more compact polymer areas.

It is worth mentioning that the wetting and the dewetting procedure shows an impressively high reversibility, and more than 10 cycles were successfully performed. This indicates that the polymer chains were strongly anchored on the surface of the gold using the strong aurophilic RAFT group, making the thin polymer film very robust for multiple dewetting cycles.

### 3.2. Self-Assembly of AuNPs upon Surfaces with Nanostructures from Constrained Dewetting

The size of the domains formed via the dewetting technique is in the same range as that of the AuNPs. By altering the molar mass of the grafted polymer as well as the grafting density, the domain sizes could be controlled between 8 and 20 nm in this work. This matching size opens up an intriguing strategy for AuNP deposition by using the nanostructured RAFT polymer as a template. In this work, two types of AuNPs were used to study the behavior of AuNP deposition towards nanostructured polymer surfaces in different solvents. Notably, 13 nm citrate-stabilized AuNPs in water and 5 nm tetraoctylammonium bromide (TOAB)-capped AuNPs in toluene provide two very distinct particle/solvent systems for the dewetted RAFT-polystyrene surfaces. Two types of experiments were conducted here: (i) To study the arrangement of AuNPs on the dewetted, nanostructured polymer film. Citrate-stabilized gold nanoparticles provide a straight-forward approach, since they are already dispersed in water, which is a suitable solvent for dewetting and for the nanostructured polystyrene film to retain its structure. (ii) In order to tightly anchor AuNPs onto the thin polymer film as a second layer, four-arm star polystyrene-functionalized gold surfaces were immersed in the sol of TOAB-capped gold nanoparticles in toluene. The particles are expected to be anchored to the surface. With a subsequent dewetting of these star-polymer/nanoparticle hybrid-layer, metallic-polymeric hybrid nanostructures with specific patterns are expected. 

**Figure 4:**
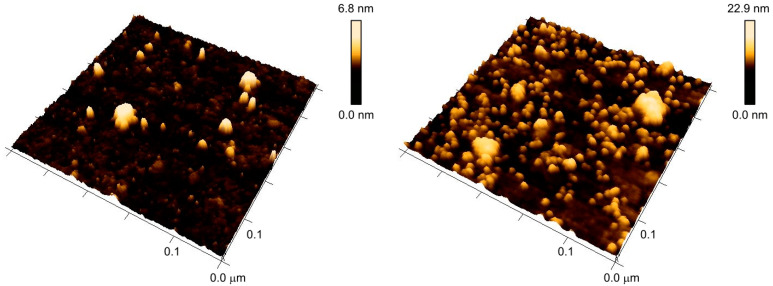
Distribution of citrate-capped AuNPs deposited on nonfunctionalized gold surfaces. AFM images show the distribution of AuNPs after one minute (**left**) and two hours (**right**) of immersion, respectively.

In addition to these experiments, a control experiment was first carried out by simply incubating a blank substrate (gold-coated glass) with AuNP-sol. First, the distribution of 13 nm citrate-stabilized AuNPs deposited on gold substrate-surfaces without any polymer brush was investigated. Gold substrates were immersed in a low concentration AuNP-sol for different time intervals, and subsequently investigated via AFM. Figure 4 shows the resulting structure after one minute of incubation in a low-concentrated (0.1 mg/mL) dispersion. Even after this very short treatment, a mixed structure of separated and aggregated gold nanoparticles could be observed (Figure 4, left). The two largest aggregates formed of several AuNPs seen in this figure have diameters of 32 nm and 45 nm, respectively. 

Clearly, a structure of irregularly-arranged nanoparticles forming clusters of different heights and feature sizes was formed. Figure 4 (right) shows an AFM image of a gold substrate that was immersed in a dispersion of gold nanoparticles for two hours. A mixed structure of separated and aggregated nanoparticles was confirmed. The number of attached nanoparticles increased, but their arrangement remained irregular, and surface-bound aggregates formed.

Then, linear RAFT polystyrene with a molar mass of *M*_n_ = 64 kg/mol was grafted onto an ultraflat gold surface. Samples with low reduced grafting densities (here Σ = 0.6) were used for dimethylformamide/water dewetting experiments. After the formation of a nanostructured polymer layer (see Section 3.1), the substrate was immersed in the aqueous sol of citrate-stabilized AuNPs.

**Figure 5:**
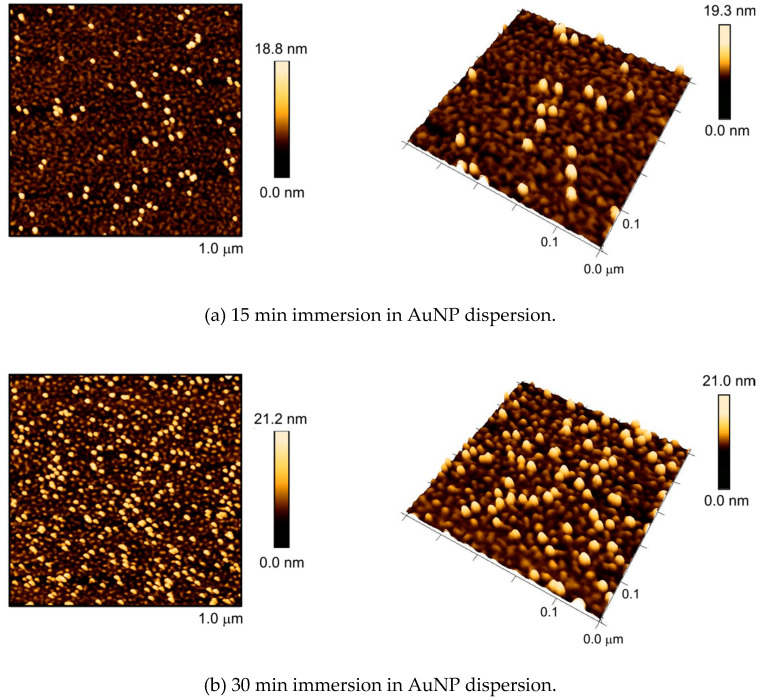

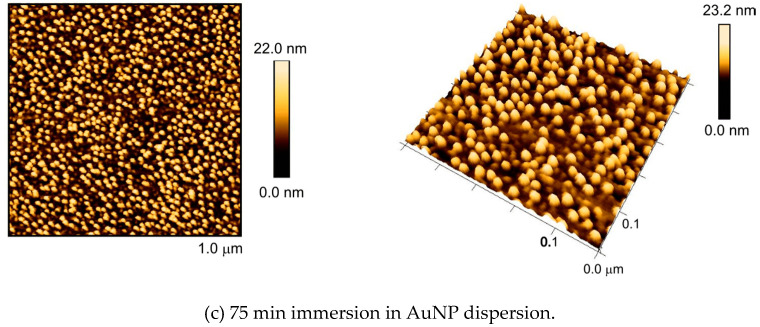
AFM images in 2D view (**left**) and 3D view (**right**) of preformed polystyrene nanostructures from the dewetting of grafted RAFT polystyrene with of *M*_n_ = 64 kg/mol on gold surfaces after different incubation times with an aqueous sol of 13 nm AuNPs.

Figure 5 shows the obtained assemblies of gold nanoparticles after different incubation times of the nanostructured surface with AuNPs. After 15 min, three percent of the surface were covered with gold nanoparticles, while the polymeric nanostructure was retained. The AFM image shows that the AuNPs were embedded in the polymeric nanostructure like eggs in an egg carton. In contrast to the substrates without the polymer layer, the nanoparticles were homogeneously distributed without any observable aggregation. The AuNPs apparently adhered to the gold surface directly, as the grafting densities are relatively low, leaving open space for particle attachment between the grafted polymer chains, but remaining separated from each other by the aid of the polymer chain. 

After 30 min of incubation with AuNPs, the surface coverage increased to ten percent. The longest treatment, i.e., 75 min, yielded very dense colloidal layers of nanoparticles where the surface coverage reached 24%. Again, in both cases, a homogeneous distribution of particles without any aggregation could be observed. It can be concluded that the polymeric nanostructure offers a powerful patterning effect for AuNPs.

**Figure 6:**
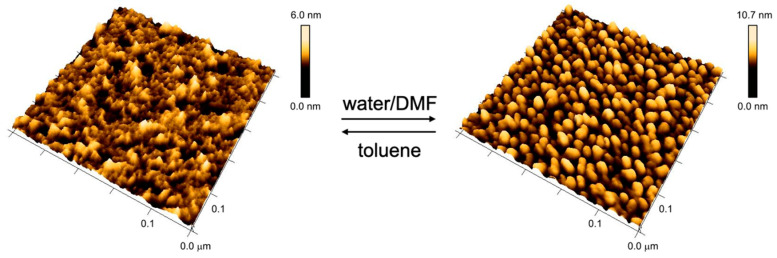
Reversible morphology switch of 5 nm AuNP–star-RAFT-polymer hybrid surface (*M*_n_ = 84 kg/mol and Σ = 2.9). On the left side, the AFM image shows the AuNP functionalized surface after incubation in toluene; no periodically-structured surface can be observed. After dewetting with water/DMF, a globular structure with ca. 21 nm domain size was formed (right). The switching of the morphology is reversible.

The method for grafting linear RAFT polymer onto the gold surface with low grafting density can also be applied to the star RAFT polymer. The star RAFT polymer on the gold surface has the ability to immobilize 5 nm AuNPs from a toluene sol, forming a secondary layer of AuNPs above the polymer layer. The dewetting protocol is then applied to the AuNP–star-RAFT-polymer hybrid surface. Again, like with grafted RAFT polymer without particles, the structured patterns can be reversibly triggered upon mixing with a poor solvent, as demonstrated in Figure 6.

Figure 6 shows the transition between the two morphologies of the AuNP–star-RAFT-polymer hybrid surface. The grafted polystyrene had a molar mass of *M*_n_ = 84 kg/mol and was grafted with Σ = 2.9. The left AFM image shows the surface of the substrate after wetting in toluene. The height difference and root mean square roughness *R*_q_ were determined to be 6 nm and 0.85 nm, respectively. For comparison, a homogeneous polymer layer typically has a height difference of approximately 2.5 nm and *R*_q_ = 0.3 nm, as, e.g., seen in Figure 3a. Both surface parameters increased here with the embedded nanoparticles, but no periodically-nanostructured surface was obtained. The AuNPs were apparently well buried in the expanded surface-bound polymer layer and could not be seen directly.

This AuNP–star-RAFT-polymer hybrid surface was then immersed in dimethylformamide, and subsequently dewetted with water. Figure 6 (right) shows a periodic globular nanostructure of polystyrene and AuNPs. The height difference and root mean square roughness increased significantly to 10.7 nm and 1.71 nm, respectively, reflecting the more pronounced structure. Furthermore, we found that the diameter of the spherical features seen on the surface was around 21 nm. It seems that individual AuNPs were apparently covered by the surface-tethered star polymer, forming a globular nanocomposite that was linked to the planar surface. It is worth mentioning that when using low molar masses of the grafted star polymer (e.g., *M*_n_ = 22 kg/mol), no regularity of AuNPs can occur. 

The dependence of the grafting density of star polymer on the dewetting of the AuNP–star-RAFT-polymer hybrid surface was studied using long-chain polymer (*M*_n_ = 84 kg/mol) with the same incubation time (24 h) but different particle sol concentrations. In this way, substrates carrying polymer brush layers having a reduced grafting density, i.e., Σ = 0.3, 1.4 and 2.9, were obtained. 

**Figure 7:**
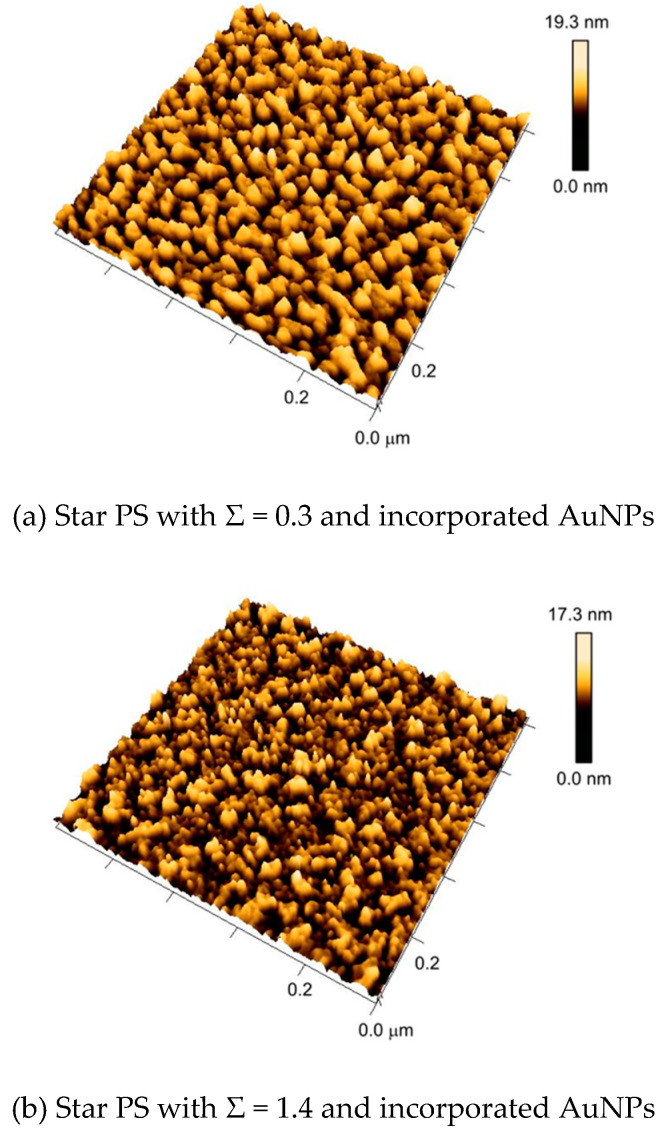

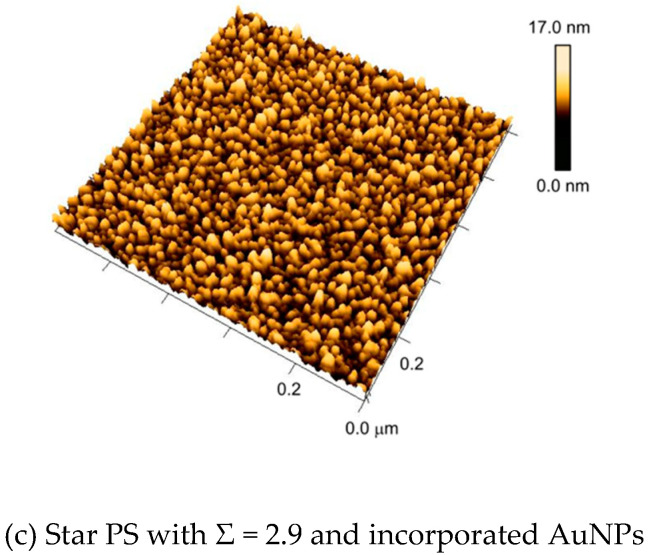
Representative AFM images of nanostructures with multicomponent assemblies of star RAFT polystyrene with *M*_n_ = 84 kg/mol and 5 nm gold nanoparticles. The reduced grafting density of polystyrene increases from (**a**)–(**c**).

Figure 7 shows the dewetted AuNP–star-RAFT-polymer hybrid surface with different star polymer grafting densities. Significant differences can be observed with increasing grafting density. The sample with the lowest reduced grafting density (Σ = 0.3) showed the largest structure domains, i.e., clearly larger than individual AuNPs. It was suspected that the AuNPs had clustered here, since there was apparently not enough polymer material to cover every individual particle. For the sample with a reduced grafting density of Σ = 1.4, a mixed structure of small and large domains was observed. It is worth mentioning that the spherical structure of the gold nanoparticles could not be observed (compared with Figure 5) directly in these cases, possibly due to the smaller size of the applied AuNPs. Figure 7c shows that only a high grafting density, where enough polymer is present, leads to the situation of more or less individual AuNPs being packed by the polymer.

## 4. Conclusions

In this work, the advantageous characteristics of the constrained dewetting technique were combined with controlled polymer design via RAFT polymerization in order to achieve a precise arrangement of AuNPs on gold surfaces at the nanoscale.

Linear RAFT polystyrene was grafted via its aurophilic RAFT end-group to a planar gold surface and subsequently dewetted to form a polymeric nanopattern. The substrate was then immersed in an aqueous sol of 13 nm citrate-stabilized AuNPs. During the incubation, AuNPs attached to the nanostructured surface and were regularly embedded in the polymer pattern, as confirmed by AFM. The number of attached AuNPs can be tuned by the incubation time of the substrate with the AuNP-sol until the formation of a dense layer of AuNPs has occurred. This approach allows uniform coverage of well-separated AuNPs on surfaces, preventing nanoparticle aggregation. Furthermore, the polymeric nanostructure offers a significant stabilization effect for AuNPs.

Star RAFT polystyrene was also grafted to gold surfaces with different grafting densities; 5 nm TOAB-capped AuNPs were immobilized to the polymer layer via attachment to the star-shaped polymer, which provides further aurophilic groups. The AuNP–star-RAFT-polymer hybrid surface also has the ability to perform dewetting-triggered morphology transition in a reversible manner, if the chain length of the star polymer chain is sufficiently long. Depending on the grafting density of the star RAFT polymer, the nanopattern of the AuNP-polymer hybrid layer can easily be varied. At high grafting densities, a well-ordered periodic hybrid layer nanopattern can be created.

## Figures and Tables

**Table 1:** Number average molar masses, *M*_n_, and dispersities, *Ð*, of the linear and four-arm star polystyrene (PS) as obtained via size-exclusion chromatography.

Reaction Time/h	*M*_n_ (Homopolymer)/kg/mol	*Ð* (Homopolymer)	*M*_n_ (Star Polymer) */kg/mol	*Ð* (Star Polymer)
6	21	1.10	22	1.13
12	40	1.08	60	1.12
24	64	1.07	84	1.11

* obtained via correcting the reduced hydrodynamic radius of star polymers [19].

## References

[1] Schmelmer U., Paul A., Küller A., Steenackers M., Ulman A., Grunze M., Gölzhäuser A., Jordan R. (2007). Nanostructured polymer brushes. Small.

[2] Minko S., Usov D., Goreshnik E., Stamm M. (2001). Environment-Adopting Surfaces with Reversibly Switchable Morphology. Macromol. Rapid Commun..

[3] Orski S.V., Fries K.H., Sontag S.K., Locklin J. (2011). Fabrication of nanostructures using polymer brushes. J. Mater. Chem..

[4] Manfrinato V.R., Stein A., Zhang L., Nam C.-Y., Yager K.G., Stach E.A., Black C.T. (2017). Aberration-Corrected Electron Beam Lithography at the One Nanometer Length Scale. Nano Lett..

[5] Liu X., Guo S., Mirkin C.A. (2003). Surface and site-specific ring-opening metathesis polymerization initiated by dip-pen nanolithography. Angew. Chem. Int. Ed..

[6] Kaholek M., Lee W.K., Ahn S.J., Ma H., Caster K.C., LaMattina B., Zauscher S. (2004). Stimulus-Responsive Poly(N-isopropylacrylamide) Brushes and Nanopatterns Prepared by Surface-Initiated Polymerization. Chem. Mater..

[7] Slim C., Tran Y., Chehimi M.M., Garraud N., Roger J.P., Combellas C., Kanoufi F. (2008). Microelectrochemical patterning of surfaces with polymer brushes. Chem. Mater..

[8] Motornov M., Sheparovych R., Katz E., Minko S. (2008). Chemical Gating with Nanostructured Responsive Polymer Brushes: Mixed Brush versus Homopolymer Brush. ACS Nano.

[9] Minko S., Usov D., Froeck C., Stamm M., Müller M., Scholl A. (2002). Lateral versus perpendicular segregation in mixed polymer brushes. Phys. Rev. Lett..

[10] Zhao B., Brittain W.J., Zhou W., Cheng S.Z. (2000). Nanopattern Formation from Tethered PS-b-PMMA Brushes upon Treatment with Selective Solvents. J. Am. Chem. Soc..

[11] Siqueira D.F., Köhler K., Stamm M. (1995). Structures at the surface of dry thin films of grafted copolymers. Langmuir.

[12] Lee T., Hendy S., Neto C. (2013). Tunable nanopatterns via the constrained dewetting of polymer brushes. Macromolecules.

[13] Huh J., Ahn C.H., Jo W.H., Bright J.N., Williams D.R. (2005). Constrained dewetting of polymers grafted onto a nonadsorbing surface in poor solvents: From pancake micelles to the holey layer. Macromolecules.

[14] Barner-Kowollik C., Davis T.P., Heuts J.P.A., Stenzel M.H., Vana P., Whittaker M. (2003). RAFTing Down Under: Tales of Missing Radicals, Fancy Architectures, and Mysterious Holes. J. Polym. Sci. Part A Polym. Chem..

[15] Ebeling E., Vana P. (2013). RAFT-Polymers with Single and Multiple Trithiocarbonate Groups as Uniform Gold-Nanoparticle Coatings. Macromolecules.

[16] Rossner C., Ebeling B., Vana P. (2013). Spherical Gold-Nanoparticle Assemblies with Tunable Interparticle Distances mediated by Multifunctional RAFT Polymers. ACS Macro Lett..

[17] Rossner C., Roddatis V., Lopatin S., Vana P. (2016). Functionalization of Planet-Satellite Nanostructures Revealed by Nanoscopic Localization of Distinct Macromolecular Species. Macromol. Rapid Commun..

[18] Tebbe M., Galati E., Walker G.C., Kumacheva E. (2017). Homopolymer Nanolithography. Small.

[19] Boschmann D., Vana P. (2007). Z-RAFT Star Polymerizations of Acrylates: Star Coupling via Intermolecular Chain Transfer to Polymer. Macromolecules.

[20] Rossner C., Vana P. (2014). Planet-satellite nanostructures made to order by RAFT star polymers. Angew. Chem. Int. Ed..

[21] Peng W., Rossner C., Roddatis V., Vana P. (2016). Gold-Planet–Silver-Satellite Nanostructures Using RAFT Star Polymer. ACS Macro Lett..

[22] Brittain W.J., Minko S. (2007). A structural definition of polymer brushes. J. Polym. Sci. Part A Polym. Chem..

